# Phase Transition-Driven Nanoparticle Assembly in Liquid Crystal Droplets

**DOI:** 10.3390/nano8030146

**Published:** 2018-03-07

**Authors:** Charles N. Melton, Sheida T. Riahinasab, Amir Keshavarz, Benjamin J. Stokes, Linda S. Hirst

**Affiliations:** 1 Department of Physics, School of Natural Sciences, University of California, 5200 North Lake Rd., Merced, CA 95343, USA; cmelton@ucmerced.edu (C.N.M.); triahinasab@ucmerced.edu (S.T.R.); 2 Chemistry and Chemical Biology Unit, School of Natural Sciences, University of California, 5200 North Lake Rd., Merced, CA 95343, USA; akeshavarz@ucmerced.edu (A.K.); bstokes2@ucmerced.edu (B.J.S.)

**Keywords:** nematic liquid crystal, quantum dot, nanoparticle, self-assembly, phase transition

## Abstract

When nanoparticle self-assembly takes place in an anisotropic liquid crystal environment, fascinating new effects can arise. The presence of elastic anisotropy and topological defects can direct spatial organization. An important goal in nanoscience is to direct the assembly of nanoparticles over large length scales to produce macroscopic composite materials; however, limitations on spatial ordering exist due to the inherent disorder of fluid-based methods. In this paper we demonstrate the formation of quantum dot clusters and spherical capsules suspended within spherical liquid crystal droplets as a method to position nanoparticle clusters at defined locations. Our experiments demonstrate that particle sorting at the isotropic–nematic phase front can dominate over topological defect-based assembly. Notably, we find that assembly at the nematic phase front can force nanoparticle clustering at energetically unfavorable locations in the droplets to form stable hollow capsules and fractal clusters at the droplet centers.

## 1. Introduction

Topological defects in nematic liquid crystals (LCs) are known to drive the self-assembly of included colloidal particles through elastic interactions with the medium [1,2,3]. Colloidal particles can be located and arranged in two- and three-dimensional packings by the action of defect lines [1] to produce custom lattice-like structures. In general, several types of self-assembled structures have been constructed in different media such as linear chains [4], clusters [5], and structured arrays [6,7]. These self-assembled structures can be driven by a variety of forces, such as kinetics of the particles themselves [8]. Two-dimensional (2D) structures composed of colloidal particles have also been formed on the surface of liquid crystal droplets suspended in water and localized at topological defects on LC droplet surfaces [9,10,11]. This has been successfully achieved using both nano- and micro-particles [12,13] as well as biological molecules [14], to achieve unique structures.

The assembly of very small particles (~10 nm in diameter or less) presents more of a challenge as the particles become subject to strong Brownian fluctuations where the size of the particles approaches that of the solvent molecules. If the energy scale of these thermal fluctuations (~kT) also becomes comparable to the free energy cost of inserting a particle into the anisotropic liquid crystal medium, spontaneous assembly mediated by the Frank elastic constants [3] can occur. Dispersed particles in the liquid crystal are able to explore the anisotropic fluid thermally and assemble at free energy minima by clustering together and/or locating in topological defect cores.

There have been several recent attempts to use liquid crystal defects to assemble and cluster nanoparticles of several types, including semiconducting (e.g., quantum dots), metallic (e.g., gold, silver), and magnetic particles [15]. Such assemblies may exhibit collective electronic, photonic, or magnetic properties not seen in isolated nanoparticles [16,17]. Typically, particle clustering experiments result in the formation of isolated aggregates with no internal ordering or position control, although the deliberate seeding of defect points [18] or lines [19] can provide an organizing mechanism. An alternative approach to forming nanoparticle assemblies uses monodisperse particles and carefully designed ligands [20]. This work has resulted in fascinating 2D arrangements of particles; however, the technique has not been expanded into large-scale structures in the third dimension.

In this work we were interested in finding a way to reliably direct the spatial organization of nanoparticle clusters and other assemblies. For example, can we make a regular array of small nanoparticle clusters, each of a well-defined size? Our approach is to use liquid crystal droplets in the nematic phase to control the positioning and size of these clusters. Micron-scale droplets containing clusters should be easy to manipulate by external methods (including, optical trapping and surface patterning).

**Figure 1:**
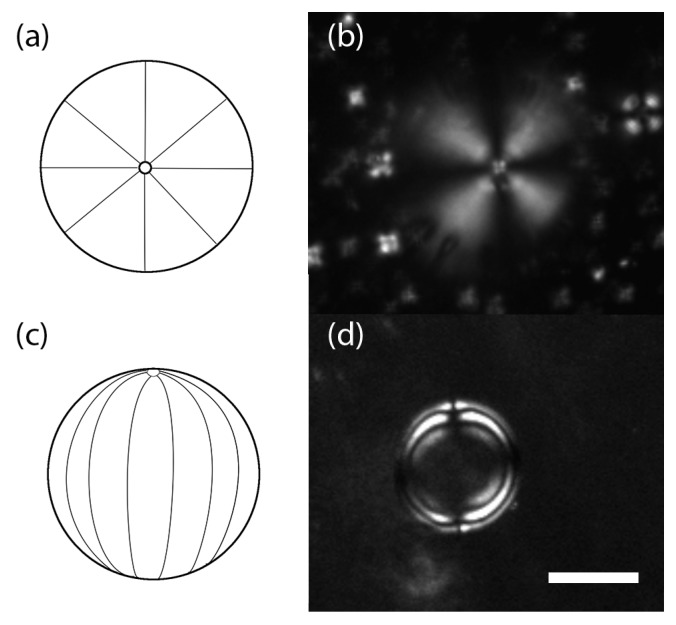
Two common director configurations for a nematic liquid crystal droplet. Radial (single defect in the center): (**a**) schematic; and (**b**) crossed polarizer image of a droplet suspended in aqueous solution, and bipolar (two surface defects); (**c**) schematic; and (**d**) crossed polarizer image of a droplet suspended in aqueous solution. Scale bar = 20 μm.

The nematic liquid crystal phase is an anisotropic fluid characterized by orientational order defined locally by the director [21]. Stable topological defects in liquid crystals occur where there is orientational frustration, for example, in bulk, at the center of a spherical nematic droplet or at the poles of a sphere coated with a thin film of smectic liquid crystal [22]. When considering a thin film of nematic liquid crystal, four +1/2 defects occur at locations that form a tetrahedron through a sphere [23]. One way to reliably control the location of these topological defects in liquid crystals is to control the geometry of the material and anchoring conditions at its interfaces. Take for example, a spherical liquid crystal droplet. Homeotropic anchoring conditions (whereby molecules are oriented perpendicular to the interface) will lead to a radial droplet structure, with a hedgehog defect located at the center (Figure 1a,b). In contrast, planar anchoring conditions (whereby molecules lie parallel to the LC/solution interface) will tend to lead to a bipolar structure with two defects located at opposite poles of the droplet (Figure 1c,d).

In previous work, large particles ranging from hundreds of nanometers to several microns in diameter were shown to pin to surface defects in liquid crystal droplets [9]. In these experiments the particle is placed near the defect and subsequently moves to the defect core. We take a different approach, by dispersing nanoparticles into droplets in the isotropic phase, and then subsequently cooling the droplets to the nematic phase. This approach is partially motivated by the inherent difficulties in manipulating individual nanoparticles. It also allows us to begin with a uniform particle distribution and observe cluster formation free from outside manipulation.

To allow spontaneous self-assembly at a defect without the influence of any external force, the particles must be very small and therefore mobile in the liquid crystal phase, taking advantage of Brownian fluctuations to locate at defect points. For this reason, we chose to work with 6-nm quantum dots (QDs) for their bright emission properties, although similar experiments using any nanoparticle type (gold, metal oxide, etc.) would be equivalent.

While self-assembly via topological defect locations is an effective strategy to pursue, recently another mechanism for spatial nanoparticle sorting in liquid crystals was developed [18,20]. Results demonstrated that the moving isotropic to nematic phase front can act as an elastic sorting mechanism for the tiny nanoparticles. Of particular interest is the formation of stable microcapsules or “shells”. These structures were formed using quantum dots with mesogenic ligands [24] providing an added degree of control in particle dispersion and cluster stabilization. When closely packed, the mesogenic ligands provide a short-range attractive interaction between nanoparticles.

In this paper we explore these two assembly mechanisms in liquid crystal droplets with different surface anchoring conditions (planar and homeotropic). These two mechanisms we title “equilibrium defect sorting” and “phase transition sorting”. By varying the droplet cooling rate through the isotropic to nematic phase transition, we observed different particle distributions in the liquid crystal droplets induced by defect locations and particle assembly at energetically unfavorable locations. In addition, we found that it was also possible to form single nanoparticle microcapsules [24] at the center of liquid crystal droplets.

Understanding the competition between the defect-based assembly and phase-transition-induced assembly is important for controlling the position of nanoparticle clusters over large length-scales without the need for chemical alignment layers or expensive lithography techniques. By investigating assembly with a controlled spherical droplet geometry, we can compare assembly mechanisms more directly, probing the effects of droplet geometry and size, as well as cooling rate, on cluster formation. In addition, we propose that clusters isolated in individual droplets can be close-packed to produce macroscopic assemblies of nanoparticle clusters in two and three dimensions.

## 2. Materials and Methods

**Scheme 1:**
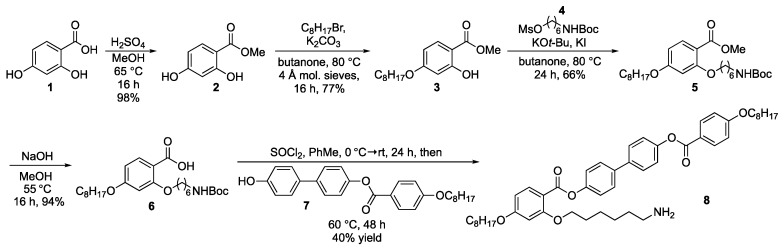
Sequence of reactions used to prepare the mesogenic ligand.

In this work we used quantum dots functionalized with a mesogenic ligand (**8**, Scheme 1). This ligand is an amine-terminated variant of the calamitic side-on attaching liquid crystals investigated by the groups of Dunmur [25] and Vashchenko [26]. It was prepared following the sequence of reactions reported by Quint and coworkers [27], and then exchanged with octadecylamine surface ligands on commercial CdSe core/ZnO shell quantum dots (NN Lab Inc., Fayetteville, AR, USA) following our reported procedure [28]. It was targeted for its ability to stabilize particle clusters via short range non-covalent interactions [19], which would facilitate particle dispersion in the host liquid crystal matrix (4-cyano-4′-pentylbiphenyl, “5CB”) and produce a uniform dispersion of modified QDs in the isotropic phase [20]. In addition, above a threshold concentration, the ligand allows the formation of micron-scale capsules [24,27] by a unique phase separation process. Herein, we aimed to form these structures in the more controlled liquid crystal droplet geometry.

The degree of ligand exchange under different conditions was quantified using ^1^H NMR spectroscopy as previously reported [28], revealing a 9:1 surface ratio of **8** to octadecylamine.

The modified particles are uniformly dispersed in 5CB via heat bath sonication at 50 °C for 2 h, verified by fluorescence microscopy. Particle concentrations used in these experiments varied between 0.05 and 0.2 wt %.

Once a uniform particle dispersion in 5CB is achieved, the droplets can be formed. We pipette 3 μL of the QD-5CB composite into 300 μL of either Millipure water or 1.0 wt % polyvinyl alcohol (PVA)/water solution at a temperature of 55 °C. 5CB droplets in water typically exhibit homeotropic boundary conditions resulting in a radial configuration which was verified by cross-polarized microscopy, whereas droplets in PVA/water solution exhibit planar boundary conditions resulting in a bipolar configuration [29], as illustrated in Figure 1c. To follow standard practice for creating nematic droplets, we also dispersed the droplets in a solution of 1 wt % sodium dodecyl sulfate (SDS) to achieve homeotropic boundary conditions, and pure glycerol to produce planar boundary conditions. After adding the QD-5CB composite to the aqueous solution, the system was then tip sonicated using a cell disrupter for approximately one second, until the resulting emulsion appeared cloudy, keeping the system above the nematic–isotropic transition temperature. The rapid motion of the tip sonicator forms droplets of varying sizes in a very small amount of time. Isotropic droplets were then cooled into the nematic phase at two different cooling rates, 1 °C/min and ~200 °C/min, and QD cluster formation and location were observed using a fluorescence microscope.

Fluorescence microscopy was used to image the spatial distribution of QDs in the liquid crystal droplets. In the experiments presented here we used CdSe/ZnS core shell QDs (NN-labs) with an emission wavelength centered at 620 nm. Fluorescence imaging was carried out on an upright Leica DM2500P microscope in reflection mode using a 20× objective. A white-light mercury lamp illumination source with a 515–560 nm band-pass filter was used for QD excitation. Emission was detected using a 580 nm dichroic mirror and a 590 nm long pass filter. The microscope can also be used in transmission mode with a white light source and crossed polarizers to image birefringence. The droplet suspensions were mounted on standard glass slides under a cover slip for observations.

## 3. Results

### 3.1. Slow Cooling Experiments

**Figure 2:**
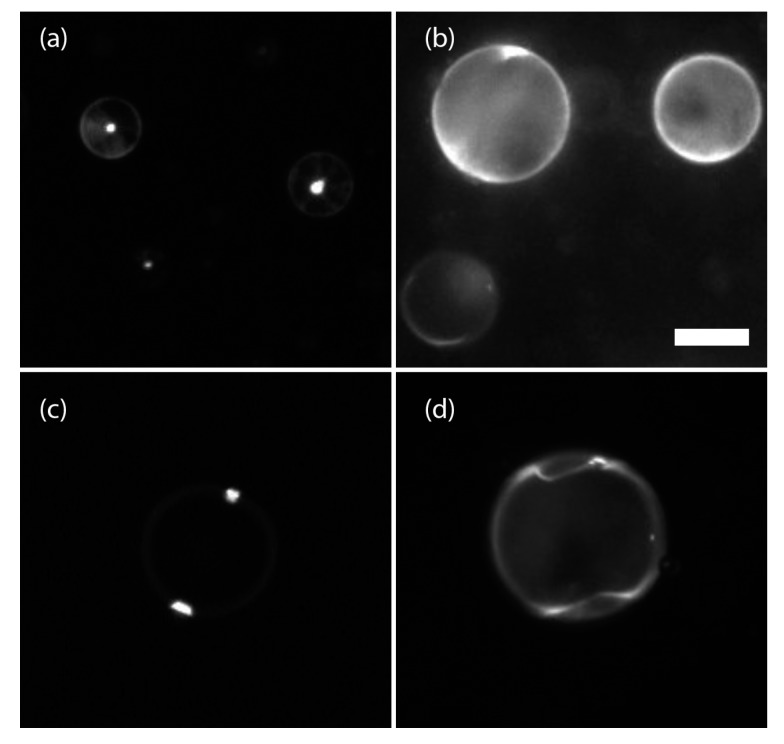
Examples of quantum dot clusters formed via slow cooling of nematic droplets. (**a**) Clusters located at the center of a droplet, with the liquid crystal in a radial configuration; (**b**,**c**) Two different examples of quantum dot distributions in bipolar droplets at a low particle concentration and (**d**) at a higher particle concentration (For all images, bar = 20 μm)

Through our experiments, we utilize two different molecular orientations for the droplets: radial and bipolar. Schematics for these molecular orientations are shown in Figure 1. In our first set of experiments, the liquid crystal droplets with dispersed QDs were cooled at 1 °C/min. Experiments were carried out at two different concentrations of QDs: 0.05 wt % and 0.2 wt %. The lower concentration was specifically chosen to prevent spherical shells and other macroscopic structures from forming via the transition templating process, as we recently reported for the same system in bulk at concentrations above ~0.15 wt % [24], and to obtain a small cluster. Figure 2 shows our results from the slow cooling experiments in which we compared radial and bipolar droplet configurations. Radial droplets resulted in QD clusters localized at the hedgehog defect at the center as shown in Figure 2a. Co-localization was verified using a combination of both fluorescence and cross-polarized microscopy. Cluster sizes varied droplet to droplet, with larger droplets producing larger central clusters. This result is expected, assuming that all droplets begin with a uniform dispersion of QDs and that these dispersed droplets all end up at the central defect after the liquid crystal transitions to the nematic phase.

### 3.2. Rapid Cooling Experiments

**Figure 3:**
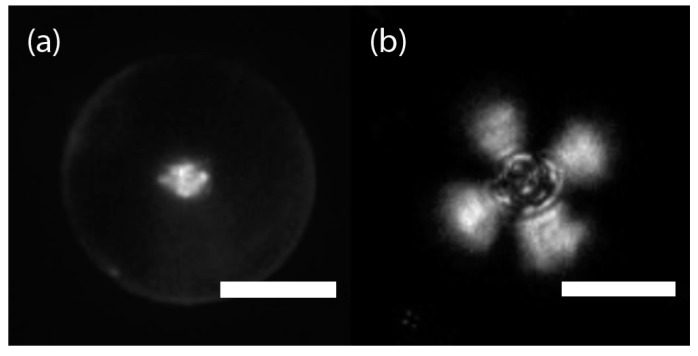
Quantum dot cluster imaged using (**a**) fluorescence microscopy and (**b**) cross-polarized bright field microscopy at the center of a droplet designed to exhibit the bipolar defect configuration. After cooling to the nematic phase, particles were found to be located at a single central point in the radial defect configuration. Scale bars = 20 μm.

In a second series of experiments we repeated the procedure described above, but with a significantly faster cooling rate of ~200 °C/min. This rapid cooling rate was chosen to match that used in recent experiments where we reported the formation of spherical QD shells by phase transition templating [24]. We first tested the low concentration QD-5CB mixture (0.05 wt %) using homeotropic boundary conditions, and again saw QD clusters forming at the center hedgehog defect. However, when droplets cooled with planar boundary conditions were examined, we observed a surprising result—the QD cluster also formed at the center (Figure 3a)—in contrast to the surface-localized particles exhibited for low cooling rates in Figure 2. Using cross-polarized microscopy, we observed that the cooled droplets in fact had radial defect conformations, not the expected bipolar conformation (Figure 2c for example). This result clearly indicates that within the appropriate parameter range, phase front sorting dominates the assembly process over the slower topological defect assembly process.

**Figure 4:**
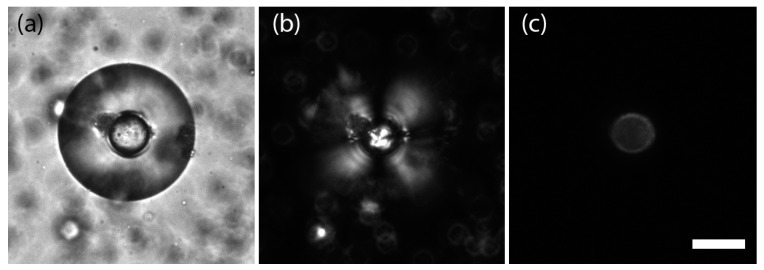
Optical microscopy images of the same droplet with a single quantum dot hollow capsule inside. (**a**) Bright-field image; (**b**) cross-polarized image; and (**c**) fluorescence image, showing quantum dots only. Scale bar = 20 μm.

We then tested the same fast cooling rate at the higher concentration of QDs in 5CB (0.2 wt %). Cooling these radial droplets produced hollow microshells located at the droplet centers. These microshells are identical to those discussed in our previous publication [24]. However, in this case, we demonstrated that it is possible to form a single hollow shell in the center of a liquid crystal droplet (Figure 4). While the previously reported bulk method for microshell formation is limited by spatial control, this new method provides a mechanism to form individual microshells at specified locations—that is, at the center of LC droplets.

### 3.3. Cluster Scaling Analysis

To quantify the clustering formation process we carried out a scaling analysis for the phase transition sorting mechanism that produced the central cluster, as shown in Figure 3. Clustering via the two different formation mechanisms produces particle packings that are quite different. Slow particle assembly by cluster-cluster aggregation and subsequent topological defect localization is expected to produce fractal-like packing with a mass-scaling dimension of 1.8 [30,31]. In contrast, the phase front templating method has been shown to produce very dense amorphous particle assemblies, including the micro-shells we demonstrated in Figure 4 [24]. A notable benefit of forming QD clusters in the confined geometry of a droplet is that it gives us the ability to quantify their spatial characteristics—since we know the concentration of particles and the droplet size, we can estimate the mass of quantum dots in each droplet. We can characterize a cluster of nanoparticles by its fractal dimension, *D*, where the relation between the mass of an object and its size is given as:

(1) $${M\;=\;\mathrm{Ar}}^{D}$$

where *M* is the mass of the object, *A* is a constant, and *r* represents the radius of the cluster. To calculate the mass-scaling dimension of nanoparticle clusters, we measured cluster size as a function of cluster mass.

In the perfect case, all particles in a droplet would be driven to the central point, and the mass of a specific cluster would simply be obtained as $M\;=\;c\frac{4}{3}{\pi R}^{3}$, where *c* is the initial particle concentration before the phase transition (in the isotropic phase) and *R* is the radius of the droplet. However, we observed that droplet images under fluorescence microscopy indicated some emission in regions other than the central defect. This leads to the conclusion that not all of the nanoparticles were swept up during the isotropic–nematic phase transition and that some remain dispersed in the nematic phase. When estimating the mass scaling dimension of our clusters, we corrected for this effect to obtain more accurate measurements of the cluster mass. To calculate a ratio of the number of nanoparticles in the cluster compared to the number of nanoparticles in the bulk droplet, fluorescence intensity was integrated over the entire droplet using ImageJ and a corrected cluster mass calculated. In addition, background fluorescence away from the droplet was measured and subtracted to account for background noise.

**Figure 5:**
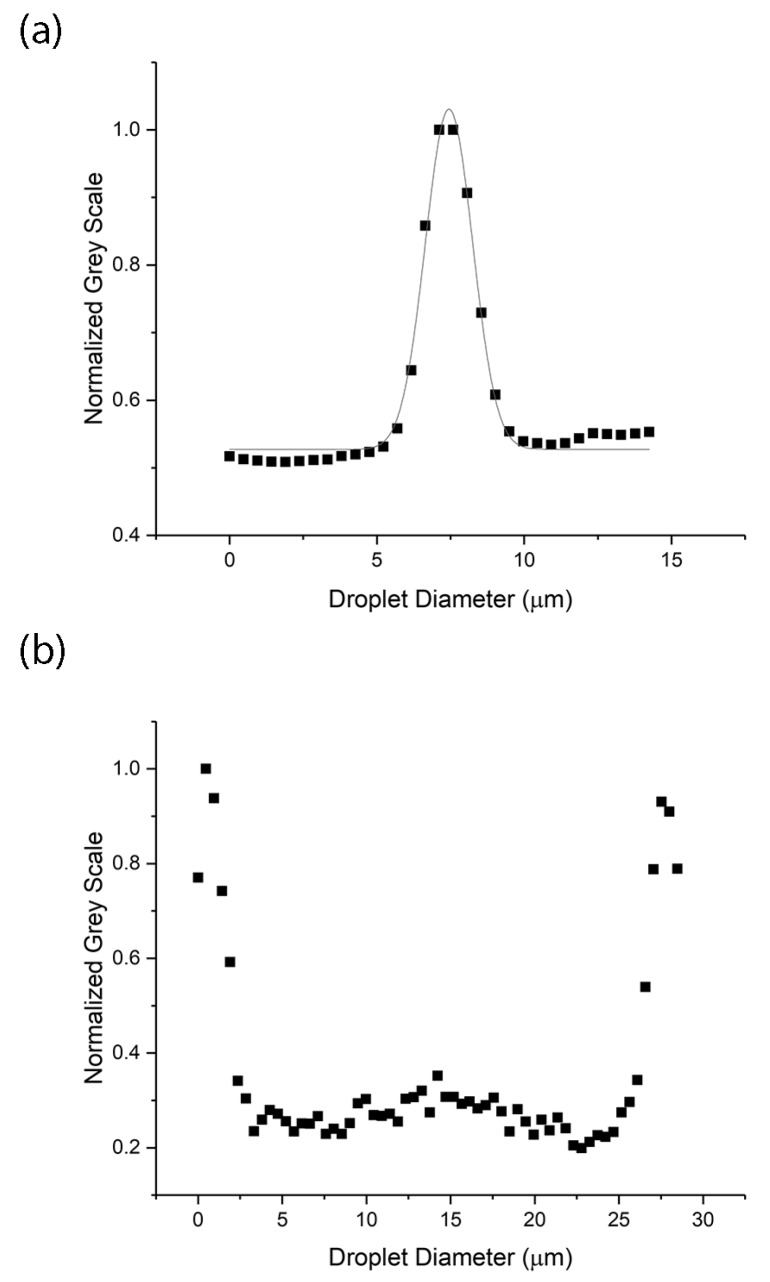
Fluorescence intensity measurements across quantum dot clusters in a droplet as determined using a one-dimensional (1D) line profile across the microscope image. (**a**) Radial droplet example measured across the central cluster (as shown in Figure 1a) showing a peak intensity at the center of a droplet with Gaussian fit; (**b**) Similar data for a bipolar droplet measured across the surface clusters (as shown in Figure 1c) showing two peak intensities at opposite poles of the droplet.

Droplet and cluster diameters were measured using bright field and fluorescence microscopy. To measure cluster diameters, three pixel-wide intensity line profiles were measured from fluorescence images and fitted with a Gaussian profile (Figure 5a). The diameter of the cluster was taken as the full-width half-max of the profile.

**Figure 6:**
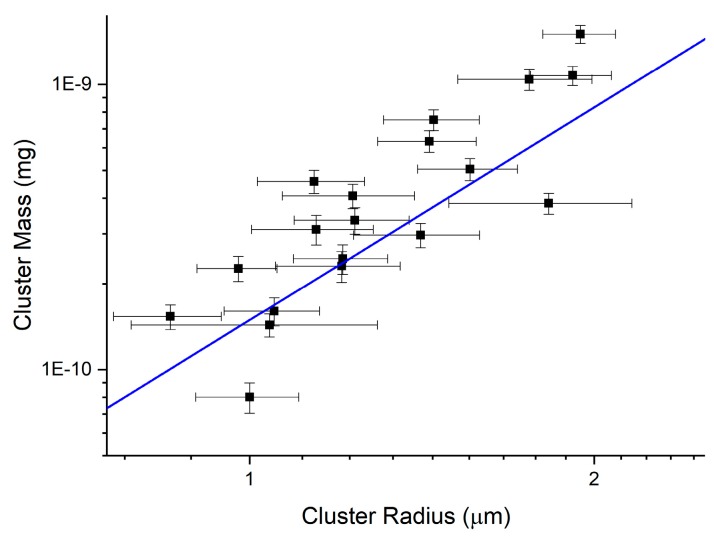
Calculated cluster mass plotted as a function of measured cluster radius. Log–log plot of Equation (1) fit to experimental data. The data collected were cluster mass and cluster size. The straight line fit on a log–log plot indicates a mass scaling dimension of 2.5 *±* 0.4.

The volume of each droplet was determined, and finally the mass of the droplet was determined using the density of 5CB. Since we know the mass of the 5CB droplet and the QD concentration by wt %, we can calculate the total mass of QDs in each droplet. With the mass and size of the quantum dot clusters, the packing fraction can be obtained. Figure 6 shows cluster mass as a function of cluster radius for an ensemble of different QD clusters formed using the phase front assembly method (rapid cooling, lower concentration). In this plot we assumed a 5% variation on the concentration of quantum dots in isotropic 5CB. The chi-square test resulted in a value of 21. Although not an excellent fit to the data, which exhibits significant scatter, this fit allowed us to quantify the linear trend, observed visually. The slope of this fit represents a scaling dimension of 2.5 *±* 0.4, which is relatively dense and consistent with the theoretical value for three-dimensional (3D) ballistic aggregation [30,31].

## 4. Discussion

Our initial hypothesis was that QD cluster patterning would be achieved through equilibrium defect formation in a liquid crystal droplet in a similar mechanism to that recently reported for micron-scale particles [10,11,12]. However, we also proposed that rapid cooling might lead to the formation and stabilization of out-of-equilibrium structures such as the recently reported QD spherical shells reported at high cooling rates in a similar system [24]. To investigate these mechanisms, we carried out two sets of experiments, focused on slow cooling and rapid cooling through the isotropic to nematic transition. The droplet geometry was particularly useful in these experiments because the ground-state topological defect configurations are well defined. In addition, the particle concentration within the droplet is easily controlled.

### Two Different Mechanisms for Assembly

In this paper we considered two separate mechanisms responsible for the spatial organization of particles in liquid crystal droplets. Overall, the collection of quantum dots occurred at the defect location (i.e., center) of the droplet by a simple free energy argument. There are three major contributions to the free energy for quantum dots inside a liquid crystal droplet: elastic forces, phase transition dynamics (Landau theory (order parameter)), and the actual insertion of quantum dots. This can be expressed as $F=F_{\mathrm{el}}+\;F_{\mathrm{LdG}}+\;F_{\mathrm{QDs}}$. The elastic free energy is the classic Frank Free energy which consists of splay, twist, and bend energies; these are the common deformations of a liquid crystal. The Landau de Gennes (LdG) free energy governs the phase transition, and the quantum dot free energy can be considered to represent the particle fraction inside the liquid crystal medium. The particles are swept up due to the changes governed by *F*_LdG_, and the location is then governed by the elastic forces that could possibly move the formed cluster to defect locations.

In the first mechanism, “equilibrium defect assembly”, the transition particles assemble in the topological defects, producing an 3D aggregate with a fractal-like mass scaling. This is consistent with our previous observations in bulk liquid crystal [20]. Given sufficient time, particles initially not located at defects will eventually migrate there by thermal motion and “fall in” to the aggregate. This process is most likely to occur at very low particle concentrations.

The second mechanism is through “phase transition assembly”. In this mechanism, recently reported by our group [20,24], particles preferentially locate in a shrinking isotropic domain within the droplet as it cools through the isotropic to nematic phase transition. The particles are pushed close together as the isotropic phase shrinks to form a cluster.

The first mechanism for nanoparticle sorting in the droplets concerns their tendency to cluster and localize at topological defects in the liquid crystal. Spherical particles of all sizes have been shown to accumulate at defect points in liquid crystals [3]. This is a relatively slow size-dependent process, whereby particles in the nematic phase tend to locate at points of low order to minimize the elastic deformation of the liquid crystal by radially symmetric particles.

The second mechanism is related to the propagation of the isotropic to nematic phase front. In this case, particles preferentially locate in shrinking isotropic domains at the phase transition and are effectively pushed together by the nucleating, growing nematic domains [20,24], entropically repelled by the ordered environment of the nematic phase. This sorting process can occur rapidly and, if the particles are small enough, provides an excellent mechanism to assemble nanoparticle-based structures.

In all of our droplets, we started with a uniform particle distribution in the isotropic liquid crystal phase. Then, following a cooling step, the liquid crystal transitioned into the nematic phase. Our results highlight several possibilities for controlling particle organization.

Using different cooling rates, we observed evidence of competition between the two different assembly mechanisms. Six-nanometer nanoparticle diffusion in the nematic phase can be generally considered as an Brownian random walk, modified by local director orientation. Particles and small clusters will explore the droplet until they locate in a topological defect. We can characterize this motion by timescale, τ_QD_, the time for a particle to travel a mean squared displacement (MSD) equal to the radius of the droplet.

As nanoparticles and small clusters of particles move randomly, they may encounter other particles or clusters, increasing their size. This will further slow their diffusion rate, increasing τ_QD,_ and thus can be described as cluster-cluster aggregation in an anisotropic environment. Slow cooling the droplet through the phase transition tends to produce many small nematic domains which nucleate and grow. At slow cooling rates this process does not appear to impact the migration of the nanoparticles to topological defects, either at the center of a radial droplet, or on the surface of a bipolar droplet (Figure 1a,c).

Rapid cooling through the phase transition allows the second assembly mechanism to dominate. In this case we typically observe a single-phase front rapidly moving across the droplet. If the phase front transition timescale, τ_PF_ (the time for the phase front to travel the radius of the droplet) is short compared to the QD clustering timescale (τ_PF_ < τ_QD_), we expect the phase front particle sorting to dominate over Brownian motion. A simple analysis supports our assumptions. The diffusion constant of a quantum dot in isotropic 5CB was estimated to be 4 μm^2^/s. This was obtained from viscosity measurements from Reference [32] and from using the Einstein–Stokes relation for diffusion, with T above the isotropic–nematic phase transition point. These values yield a linear diffusion length for the QDs of ~5 μm/s in a 3D droplet. To quantify the phase front timescale (τ_PF_), we analyzed a 60-μm diameter droplet and measured the phase front velocity to be 27 μm/s. These numbers illustrate that we can expect the phase front to move approximately five times faster than the linear diffusion of an average QD in the nematic phase.

We can relate the phase transition assembly process to a non-quasistatic compression. In the case that phase transition assembly dominates, the particles are not able to diffuse away before being swept up by the phase boundary. The particles then follow the direction of the phase front, which depends on the temperature gradient direction in the liquid crystal droplet. If all of the particles are swept up, and the cluster is of sufficient size compared to the size of the droplet, Frank elastic effects are insufficient to move the large cluster, and so the liquid crystal will reorient itself around the cluster to minimize the free energy of the system. This reorientation may not always be the lowest energy state possible, as seen with the surprising example of a cluster stabilized at the center of a bipolar droplet (Figure 3). This phase front aggregation process could be described as a ballistic diffusion-limited aggregation process as particles are pushed together in the shrinking isotropic domain.

## 5. Conclusions

In this paper we investigated the spontaneous assembly of nanoparticle clusters in the confined environment of a liquid crystal droplet using mesogen-functionalized quantum dots. Varying surface anchoring conditions at the droplet/water interface allowed us to tune between radial and bipolar topological defect configurations. By tuning particle concentration and cooling rate across the isotropic to nematic phase transition, it was possible to observe the competition between two distinct assembly mechanisms: equilibrium defect assembly and phase transition assembly. We observed two key effects. First, slow cooling allows for ground state defects to template QD cluster formation, while fast cooling allows for the isotropic–nematic phase boundary to template the clusters. Secondly, we noticed that phase transition templating can be used to force non-equilibrium defect configurations, such as a radial director distribution, despite initial bipolar anchoring conditions. This droplet technique also provides a method to control quantum dot micro-shell (and cluster) formation location, opening up the possibility for easy spatial control of micron-scale nanoparticle assemblies.
